# Association between depression severity, mental health recovery and dropout from behavioral health care treatment

**DOI:** 10.1016/j.psycom.2024.100185

**Published:** 2024-07-16

**Authors:** Jason B. Gibbons, Shelbi A. Cox, Loreen Straub, Josephine S. Au, Philip S. Wang, Jun Liu, Alyson Albano, Rachel Wood, Matthew Ruble, John Peloquin, Rajendra Aldis, Lauren V. Moran

**Affiliations:** a Department of Health Systems, Management and Policy, Colorado School of Public Health, University of Colorado Anschutz Medical Campus, Aurora, CO, USA; b Discovery Behavioral Health, Irvine, CA, USA; c Division of Pharmacoepidemiology and Pharmacoeconomics, Brigham and Women’s Hospital, Harvard Medical School, Boston, MA, USA; d Department of Psychiatry, Cambridge Health Alliance, Harvard Medical School, Boston, MA, USA; e Department of Psychiatry, Mclean Hospital, Harvard Medical School, Boston, MA, USA

## Abstract

**Background:**

Understanding the relationship between depression severity, patient recovery, and treatment continuity may help optimize the delivery of behavioral health services.

**Methods:**

Using Cox proportional hazards regression, this study measures the association between treatment dropout and baseline depression severity, as measured by the Patient Health Questionnaire (PHQ-9), and the association between treatment dropout and baseline recovery, as measured by the Recovery Assessment Scale (RAS). The study also explores heterogeneity by service line (general mental health, substance use disorder, and eating disorder), residential versus intermediate level of care setting, age groups (adolescent and adult), and dropout factors. The data include 14,689 patients treated at a multi-state behavioral health care provider, discharged between 2021 and 2022. Premature dropout from behavioral health care treatment for any cause, patient factors and administrative factors were used as separate outcomes.

**Findings:**

A unit increase in baseline PHQ-9 was associated with a 1.2% reduced treatment dropout risk (hazard ratio (HR): 0.988; 95% confidence interval [0.983–0.992]). A unit increase in baseline RAS score was associated with 0.5% increased dropout likelihood (HR: 1.005; 95% CI [1.004, 1.007]). Subgroup analyses show associations are driven by general mental health, adolescent, and intermediate level of care subgroups. Patients with higher baseline suicidality and lower willigness to ask for helphad a reduced risk of dropout.

**Interpretation:**

Patients with greater depression severity and lower recovery scores at admission were more likely to stay in behavioral health treatment, especially among adolescents, patients with general mental health issues, and outpatients.

## 1. Introduction

Treatment dropout has been estimated to affect approximately 20% of patients who receive mental health treatment (Edlund et al., 2002; Swift and Greenberg, 2012; Wang, 2007). Among those who terminate their mental health care early, over 70% do so after their first or second visit (Olfson et al., 2009). Premature dropout disrupts patient treatment plans, which can lead to poor health outcomes (Cahill et al., 2003; Schindler et al., 2013; Ogrodniczuk et al., 2005). For instance, treatment dropout by patients with a substance use disorder has been found to increase relapse risk, while dropout for patients with depression has been linked to lower remission rates (Lappan et al., 2020; Onken et al., 1997; Warden et al., 2009).

There are many factors associated with mental health treatment dropout. Patient characteristics previously associated with mental health treatment dropout in other research include being a member of a racially/ethnically minoritized group (Wang, 2007; Lappan et al., 2020; de Haan et al., 2018; Green et al., 2020; Reis and Brown, 1999; Wang et al., 2009), female sex assigned at birth, (Wang et al., 2000) lower levels of education, (Olfson et al., 2009; Wang et al., 2000) low income, (Olfson et al., 2009; Warden et al., 2009; Reis and Brown, 1999) inadequate health insurance coverage, (Edlund et al., 2002; Olfson et al., 2009; Wang et al., 2000, 2009) and age (Edlund et al., 2002; Swift and Greenberg, 2012; Wang, 2007; Warden et al., 2009; Wang et al., 2009). Patient comorbidity has also been associated with premature treatment dropout, especially comorbid substance use disorder, (Wang, 2007; Olfson et al., 2009; Roseborough et al., 2016) eating disorder, personality disorder, (Swift and Greenberg, 2012) or obsessive-compulsive disorder (Roseborough et al., 2016). Psychosocially, patients wanting to handle problems independently is one of the most robust predictors of treatment dropout (Britt et al., 2015; Hoge et al., 2014; Kessler et al., 2001; Mojtabai et al., 2011). Other psychosocial factors found to be associated with dropout include improvements in mental health, stigma, (Britt et al., 2015; Hoge et al., 2014; Mojtabai et al., 2011) believing that mental health treatments are not effective, feeling uncomfortable in mental health care, having low perceived need, (Edlund et al., 2002; Hoge et al., 2014) and having negative experiences with providers (Mojtabai et al., 2011).

Research on behavioral health severity and treatment dropout presents mixed findings. Several studies have pointed to higher symptom severity at the time of dropout or during care as significant predictors of dropout (Zieve et al., 2019; Binnie and Boden, 2016; Jarrett et al., 2013; Primm et al., 2000). This is similar to results from a study that showed that patients with more severe disorders reported more reasons to drop out from treatment (Edlund et al., 2002). However, in a study of therapy attendance following a referral from a general practitioner, patients with lower non-risk scores on the Clinical Outcomes in Routine Evaluation-Outcome measure (i.e., less emotional distress) were less likely to attend their first treatment sessions than those with higher scores (Di Bona et al., 2014). Another study on dropout from psychotherapy found a statistically insignificant relationship between the psychological component of the Global Assessment of Functioning score and treatment dropout (Fenger et al., 2011).

To improve patient engagement in behavioral health care, additional evidence is needed on the relationship between clinical severity, recovery, and treatment dropout. Studies on behavioral health illness severity typically utilized small patient samples across multiple clinical conditions. Existing research has also been limited in considering different reasons for treatment dropout and often did not control for or address differences in patient treatment duration or historical treatment episodes. This study addresses limitations in existing research on behavioral health severity and treatment dropout using a large sample of patients treated by a national US behavioral health care provider. In addition to depression severity, we studied the relationship between patient recovery and dropout. We also investigated whether associations vary across patients of different age cohorts, mental health conditions, treatment settings, and dropout types (patient versus administrative-related). The latter is a particularly novel area of inquiry; organizational factors (e.g., closure due to COVID-19, insurance denials) likely differ strongly from patient-related factors (e.g., patient care dissatisfaction) given the lack of patient ability to control organization-based decisions. However, most studies leveraging dropout as an outcome measure do not distinguish between them. Finally, we evaluated specific items within our behavioral health severity and recovery measures to determine the strongest predictors of dropout.

## 2. Methods

### 2.1. Data

Our primary data source was 2021–2022 electronic health records (EHR) from Discovery Behavioral Health (DBH). DBH is a mental health treatment provider operating 154 treatment facilities across 16 states, serving roughly 14,000 patients annually. Our data included a cohort of patients receiving care at DBH’s general mental health, eating disorder, or substance use disorder treatment programs from 132 treatment facilities across 15 states from 2021 to 2022. The EHR captured patient demographics (i.e., age, race, ethnicity, and sex assigned at birth), mental health diagnoses, aggregate and individual item scores for PHQ-9 and RAS, treatment episode length, the number of past treatment episodes within DBH, whether treatment was provided at a residential treatment center (RTC) or intermediate level of care program (i.e., intensive outpatient, or partial hospitalization program), the service line of the facility where the patient received treatment (i.e., mental health, eating disorder, or substance use disorder), and the type of discharge (e. g., against treatment advice, administrative discharge, transfer, etc.).

### 2.2. Study population

Our initial study population included all patients at DBH who were administered a PHQ-9 and RAS at the time of admission between 2021 and 2022, which consisted of 17,679 patients. From this initial sample, we excluded 402 individuals with a missing or unknown discharge type. From the post-exclusion sample of 17,277 patients, we excluded 2,588 more patients whose admission or discharge PHQ-9 or RAS assessment occurred outside of a 3-day window from the admission or discharge date. This exclusion was necessary to ensure the assessments were specific to the admission or discharge time point and consistent with DBH protocols. From our final sample of 14,869 patients, we created cohorts for subgroup analyses by DBH line of service (i.e., mental health, eating disorders, and substance use disorders), age group (i.e., adolescents and adults), and program type (i.e., residential treatment and intermediate level of care).

### 2.3. Outcomes

The primary outcome in our study was treatment dropout for any reason. We captured this outcome using an indicator variable set to one if the discharge reason listed was among 1) against treatment advice, 2) administrative discharge, 3) insurance denial, 4) pandemic-related concerns, and zero if the discharge reason was among 1) completed treatment, 2) higher level of care needed, or 3) transfer. We also created two additional outcomes: treatment dropout for patient-related causes and treatment dropout for administrative causes to see if the dropout reason modified our associations. Treatment dropout for patient-related causes was represented by an indicator set to one if the discharge reason was against treatment advice. In contrast, treatment dropout for administrative causes was an indicator set to one if the discharge reason was among administrative discharge, insurance denial, or pandemic-related concerns. We excluded observations for the unused outcome in models using these reason-specific outcomes.

### 2.4. Exposures

Our primary exposures were total PHQ-9 and RAS scores measured at admission. PHQ-9 is a continuous measure ranging from 0 to 27, with higher scores indicating greater depression severity. Each item ranges from zero (i.e., not at all) to three (i.e., nearly daily). RAS is a 41-item mental health recovery scale ranging from 41 to 205, with higher scores indicating more significant self-reported recovery. Each item ranges from one (i.e., strongly disagree) to five (i.e., strongly agree). Due to their potential collinearity, we analyzed the relationship between each scale and dropout in separate models.

### 2.5. Covariates

Our analyses adjusted for several patient characteristics that could confound our results. Covariates included patient age, race, ethnicity, sex assigned at birth, and primary diagnosis at admission (i.e., see Table 1 for the list of conditions), care setting (i.e., RTC or intermediate level of care), total number of mental health diagnoses, treatment episode number, and length of treatment in days. Mental health diagnoses were identified using ICD-10 F codes.

### 2.6. Statistical analysis

We used a Cox proportional hazards regression model with patient-level random effects (i.e., “frailty model”) to measure the association between PHQ-9 depression severity and treatment dropout and RAS recovery and treatment dropout. Our analyses followed patients from admission and censored them at treatment discharge or dropout.

In the model using dropout for any reason as an outcome, we started by including RAS or PHQ-9 at baseline without other covariates (i.e., unadjusted models). We then adjusted for pre-defined covariates in each model to address potential confounding. Finally, we repeated each analysis using dropout due to patient-related factors and dropout due to administrative factors as outcomes.

Next, we reran each model for the three primary DBH lines of service, age groups, and care settings for subgroup analyses. As a sensitivity analysis, we performed subgroup analyses by length of stay. Specifically, we divide patients into three lengths of stay groups: *<*30 days length of stay, 30–60 days length of stay, and 60+ days length of stay to assess whether the length of stay modified our main findings.

In addition to using the total RAS and PHQ-9 scores, we estimated our primary models using each of the five factors for the RAS and the individual items for the PHQ-9 to determine the factors most strongly associated with treatment dropout. We used the five factors of the RAS instead of all 41 items, as previous research has shown that the RAS is best explained by a five-factor model representing different facets of recovery: reliance on others, goal and success orientation, willingness to ask for help, personal confidence and hope, and no longer dominated by symptoms (Corrigan et al., 2004).

## 3. Results

### 3.1. Characteristics of DBH patients

Table 1 summarizes the characteristics of our analytic cohort for the entire sample. In the full sample, we found that DBH patients were primarily adults (57.51%), female (66.00%), non-Hispanic (62.99%), and white (69.39%). Distributions by DBH service lines were similar; the substance use disorder service line serves the most patients (37.81%) with alcohol use disorder (21.44% of total patients) as the most common primary diagnosis; the mental health service line serves 35.45% of DBH patients with depression (25.28% of total patients) as the most common primary diagnosis; the eating disorder service line serves 26.74% of DBH patients with anorexia nervosa (17.29% of total patients) as the most common primary diagnosis. The average number of patient mental health diagnoses at admission was 3.01 (standard deviation of 1.41). The 14,689 patients in the full sample had 17,580 treatment episodes (i. e., 1.26 on average per patient). These episodes mainly occurred in residential settings (56.96%), with an average length of stay of 38.07 days across all settings (standard deviation of 30.92). Average PHQ-9 and RAS at baseline were 13.29 (standard deviation of 7.43) and 149.96 (standard deviation of 24.50), respectively.

Table 1 also compares patients who dropped out of treatment for any reason (N = 3,867) against those who did not drop out of treatment (N = 10,822). Relative to patients who did not drop out of treatment, patients who dropped out were more likely to be adults, Hispanic or Latino, Native Hawaiian or Pacific Islander, received care from the mental health service line or the eating disorder service line, and treated in the intermediate level of care setting. Patients who dropped out also had shorter lengths of stay (25.79 days versus 31.20 days), lower baseline PHQ-9 scores (12.69 versus 13.50), and higher baseline RAS scores (152.02 versus 24.31) than non-dropout patients.

In our patient dropout sample, we observed 2,234 patients across 2,614 episodes. Our administrative dropout sample accounted for 1,633 patients across 1,983 treatment episodes. Patient characteristics differences between the non-dropout sample, the administrative dropout sample, and the patient dropout sample were similar to differences between the combined dropout sample and the non-dropout sample (See Table 1 and Appendix Table 3).

### 3.2. Hazard Ratio Estimates from PHQ-9 models

**Fig. 1.**
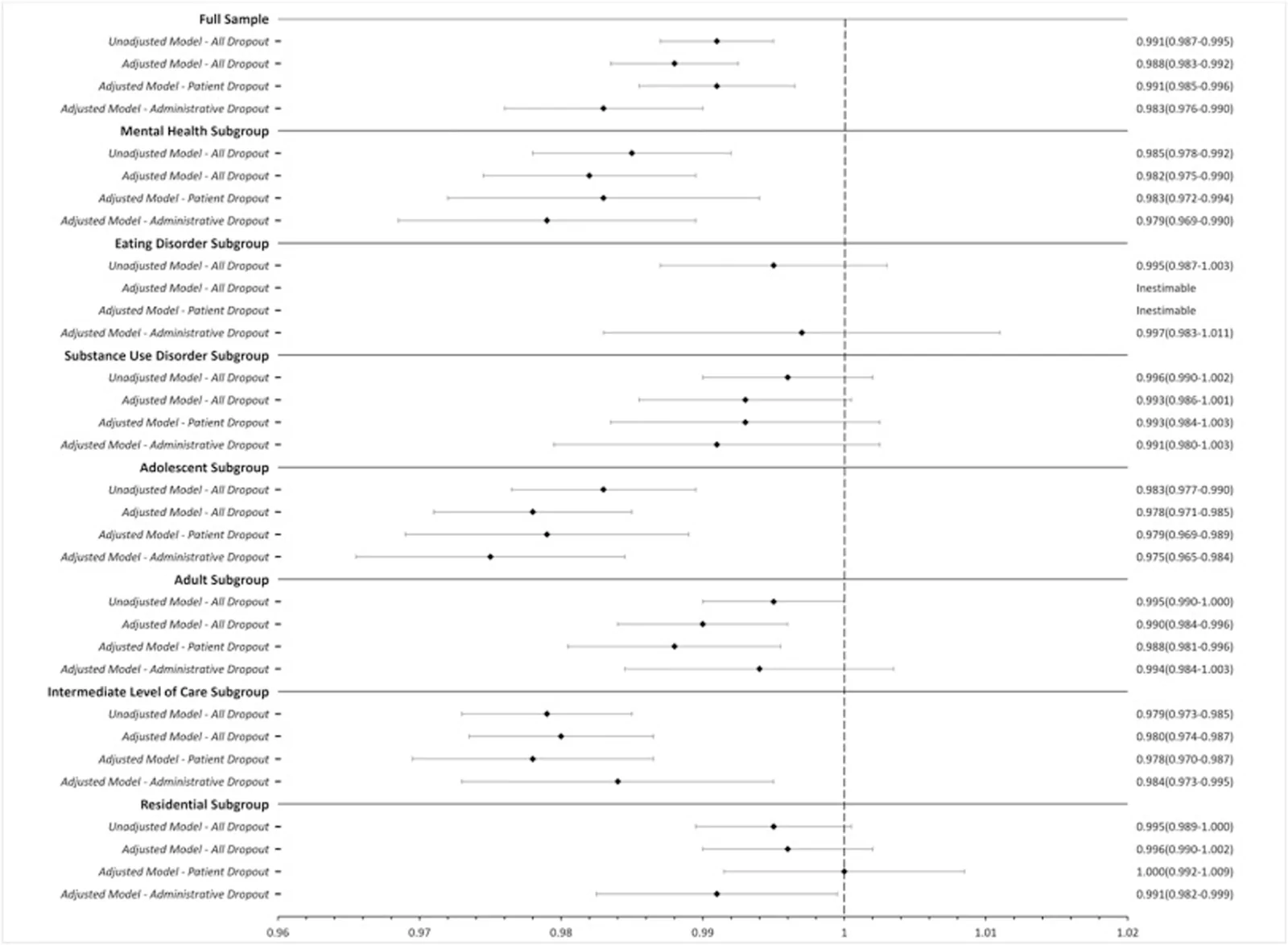
Forest plot of hazard ratio estimates from PHQ-9 models. Notes: The figure displays model estimates from the PHQ-9 treatment dropout analyses. Models include unadjusted for the all-dropout outcome, covariate-adjusted for the all-dropout outcome, adjusted for the patient dropout outcome, and adjusted for the administrative dropout outcome. Models were run for the full sample and cohort subgroups. The intermediate level of care reflects outpatient, intensive outpatient, and partial hospitalization. All estimates are presented as Hazard Ratios with 95% confidence intervals. Source: Discovery Behavioral Health Electronic Health Records Data 2021–2022.

Fig. 1 reports hazard ratios and corresponding 95% confidence intervals from the PHQ-9 analyses for the unadjusted model using all dropouts as an outcome and covariate-adjusted models with outcomes of all dropouts, patient-related dropouts, and administrative-related dropouts (i.e., the four primary models). Each model identified an inverse or statistically insignificant association between baseline PHQ-9 and treatment dropout in the full sample. Each unit increase in PHQ-9 (i.e., greater depression severity) was associated with a 0.9–1.7 percent reduction in treatment dropout depending on the reason for dropout and covariate adjustment. The direction and magnitude of this relationship were similar throughout each subgroup analysis, but not all PHQ-9 estimates were statistically significant. Significant inverse associations were found between PHQ-9 and dropout in the mental health, intermediate level of care, and adolescent subgroups for all three dropout types. Patient dropout was inversely associated with adult dropout for any reason and patient-related dropout. Decreased dropout for each unit increase of the PHQ-9 was higher in the adolescent vs. adult subgroups. Only administrative dropout was associated with PHQ-9 in the residential subgroup.

### 3.3. Hazard Ratio Estimates from RAS models

**Fig. 2.**
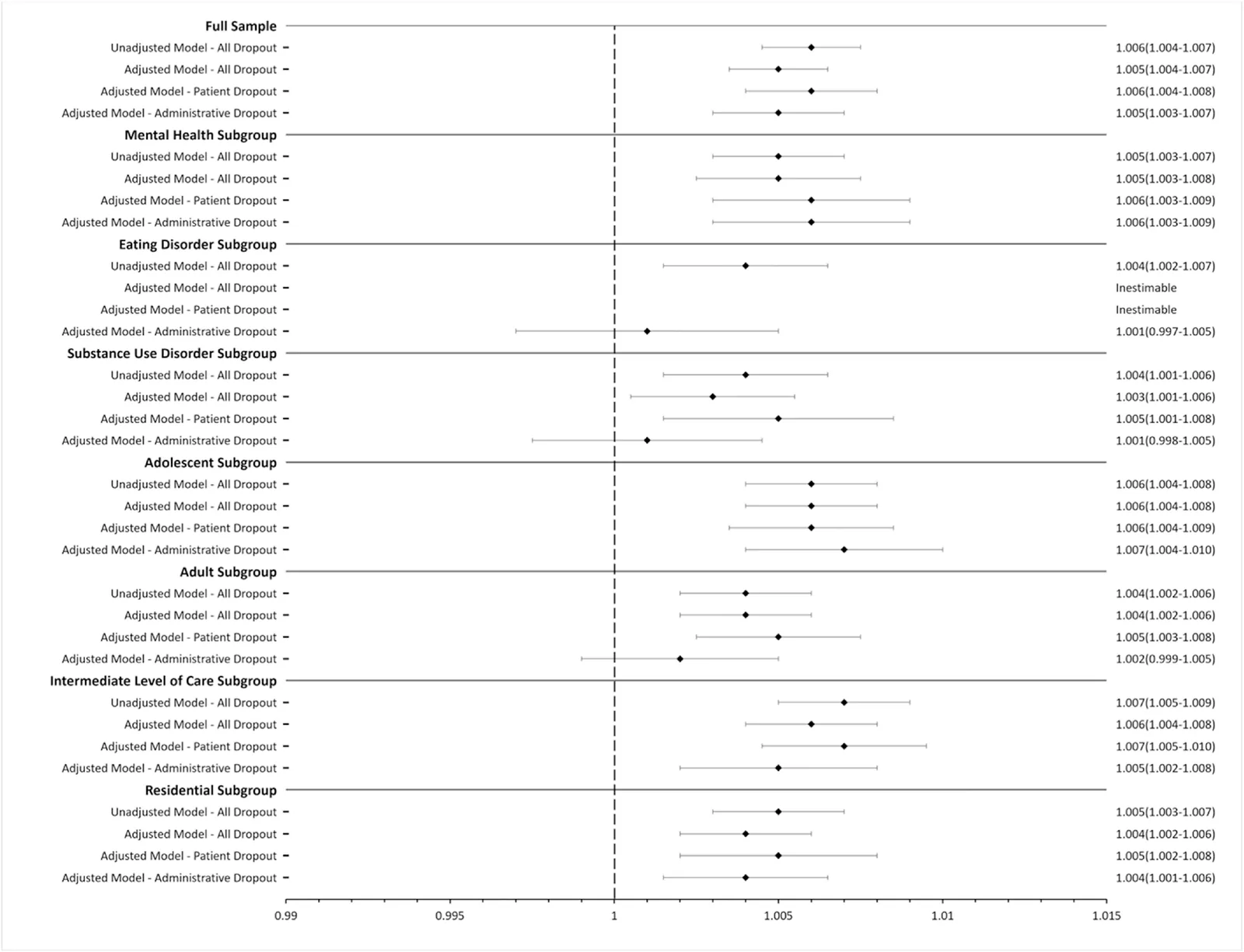
Forest plot of hazard ratio estimates from RAS models. Notes: The figure displays model estimates from the RAS treatment dropout analyses. Models include unadjusted for the all-dropout outcome, adjusted for the alldropout outcome, adjusted for the patient dropout outcome, and adjusted for the administrative dropout outcome. Models were run for the full sample and cohort subgroups. The intermediate level of care reflects outpatient, intensive outpatient, and partial hospitalization. All estimates are presented as Hazard Ratios with 95% confidence intervals. Source: Discovery Behavioral Health Electronic Health Records Data 2021–2022.

Fig. 2 reports hazard ratios from the RAS analyses for the entire sample and by subgroup. In the whole sample, each unit increase in baseline RAS (i.e., greater baseline RAS recovery levels) increased the dropout risk by 0.5%–0.6%. As with the PHQ-9 analyses, the direction and magnitude of the RAS finding were similar across all subgroups, with only notable differences in statistical significance. Specifically, positive associations were identified between RAS and dropout for the three dropout types in the mental health, adolescent, intermediate level of care, and residential subgroups. In the eating disorder subgroup, RAS was positively associated with all dropout only. In the substance use disorder and adult subgroups, RAS was positively associated with all dropouts and patient dropouts.

### 3.4. Top five PHQ-9 items associated with treatment dropout

**Fig. 3.**
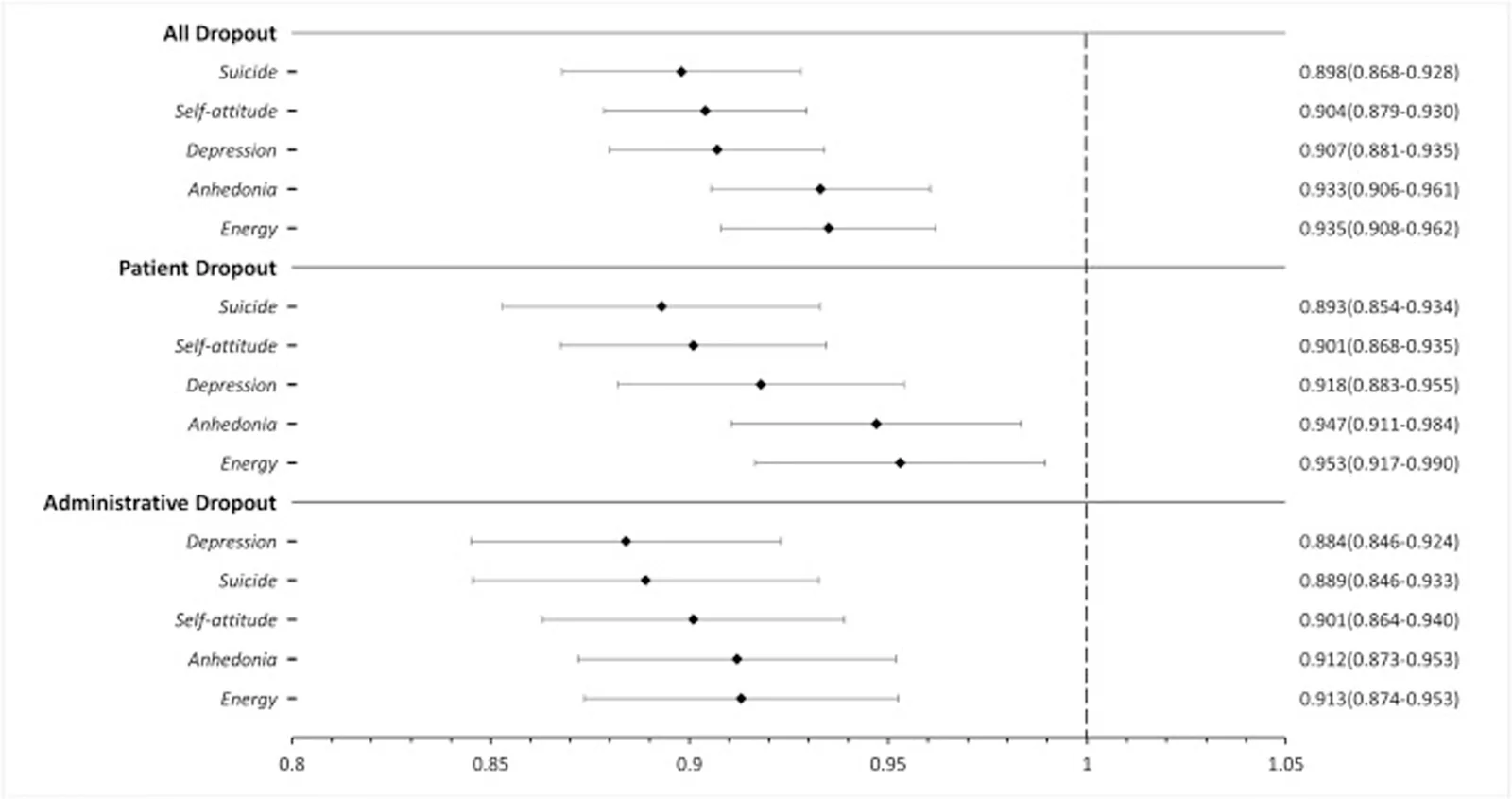
Forest plot of the top five PHQ-9 factors associated with treatment dropout. Notes: The figure displays the top five factors from the PHQ-9 associated with treatment dropout for the different models. Models include covariate-adjusted for all dropout outcomes, covariate-adjusted for the patient dropout outcome, and covariate-adjusted for the administrative dropout outcome. Models were run for the full sample. All estimates are presented as Hazard Ratios with 95% confidence intervals. Self-attitude refers to PHQ-9 question 6 (“feeling bad about yourself”). Source: Discovery Behavioral Health Electronic Health Records Data 2021–2022.

In Fig. 3, we present the five items from the PHQ-9 with the strongest associations with all dropouts, patient-related dropouts, and administrative-related dropouts for the covariate-adjusted models. As with the PHQ-9 aggregate score measure, a unit increase in the response type (0: not at all, 1:several days, 2: more than half the days, 3: nearly all the days) for individual items was associated with reductions in dropout of all types. For all dropout and patient dropout outcomes, suicidal ideation/thoughts of self-harm showed the most robust associations, where higher scores on suicidal ideation were associated with decreased dropout. For administrative dropout, depression was the most empirically significant factor, reducing dropout by nearly 12%.

### 3.5. Associations of five factors of RAS and treatment dropout

**Fig. 4.**
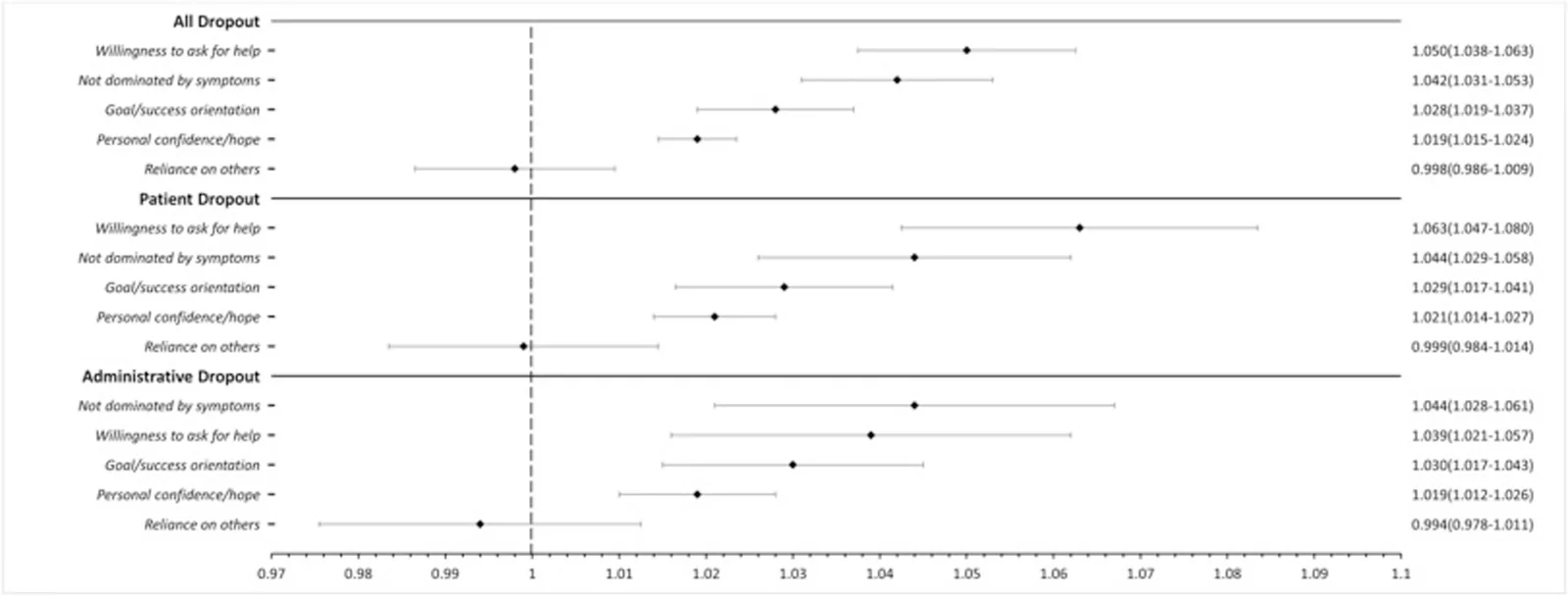
Forest plot of the association of RAS factors with treatment dropout. Notes: The figure displays the five factors from the RAS associated with treatment dropout for the different models. Models include covariate-adjusted for the all dropout outcome, covariate-adjusted for the patient dropout outcome, and covariate-adjusted for the administrative dropout outcome. Models were run for the full sample. All estimates are presented as Hazard Ratios with 95% confidence intervals. Source: Discovery Behavioral Health Electronic Health Records Data 2021–2022.

In Fig. 4, we present the associations between the five-factor RAS with all dropouts, patient-related dropouts, and administrative-related dropouts for the covariate-adjusted models. As with the RAS total score measure, a unit increase in the response type for each factor was associated with significant increases in dropout of all types or was not statistically significant. Higher scores for willingness to ask for help and not being dominated by symptoms held the most robust associations with all three dropout types. However, the order was reversed for administrative dropout (i.e., not dominated by symptoms and then willing to ask for help).

### 3.6. Sensitivity analysis

Our sensitivity analyses show that associations between baseline PHQ-9 and RAS and treatment dropout for the 0–30-day treatment duration group and the 31–60-day treatment duration group were similar to the main estimates (See Appendix Tables 1 and 2). However, all estimates for the 60+ day group across each measure were statistically significant.

## 4. Discussion

### 4.1. Interpretations of findings

Our findings suggest that patients with higher baseline PHQ-9 scores (i.e., greater depression severity) are more likely to stay in treatment than patients with lower baseline scores. They also demonstrate that patients with lower baseline RAS scores (i.e., lower self-reported recovery) are more likely to remain in treatment than patients with higher baseline scores (greater recovery). These results imply that patients in greatest need of treatment are more likely to stay in treatment than patients with less severe depression or greater RAS recovery scores. This finding is supported by studies that find dropout may be more likely among people with a lower perceived need for treatment (Edlund et al., 2002; Hoge et al., 2014) and a separate study finding lower emotional distress at baseline increased the likelihood patients would not attend their first therapy session (Di Bona et al., 2014). Our results are also consistent with prior studies that patients wanting to handle problems independently strongly predicted dropout; higher scores on RAS factors, such as willingness to ask for help at baseline, were associated with higher dropout. Patients with higher RAS factor scores may be more likely to dropout due to mismatch between patient expectations and treatment provision or higher confidence that they can handle problems independently. For the PHQ-9, the most substantial relationship for individual items was suicidality, where high levels of suicidality were associated with decreased all dropout (i.e., combined patient and administrative dropout) and patient-related dropout risk. Patients with high levels of suicidality face substantial severity and clinical risk, and so their stronger likelihood of being retained in treatment is a particularly important finding. Our subgroup analyses suggest that the mental health service line and adolescent and intermediate-level care groups primarily drive our association in the full sample results. Interestingly, no association was identified between the PHQ-9 or the RAS and treatment dropout for any models in the substance use disorder subgroups, and only baseline RAS was associated with treatment dropout in the eating disorder subgroup. Moreover, for PHQ-9 in the residential subgroup, we did not find an association with treatment dropout, but for RAS, we did find an association. The main findings were also close in magnitude and significance to results from our sensitivity analyses for short and medium stay lengths (i.e., 0–30 days and 31–60 days). However, no estimates were significant for patients with stays longer than 60 days, possibly due to fewer patients with these lengths of stay.

Although some evidence in the literature supports our findings, other studies have previously found that greater depression severity strongly predicted dropout (Edlund et al., 2002; Zieve et al., 2019; Binnie and Boden, 2016; Jarrett et al., 2013; Primm et al., 2000). Our use of baseline severity measurements, rather than changes in depression severity across treatment, may partly explain differences. We also have a population with significant severity and comorbidity, given average baseline PHQ-9 and RAS scores of 13.29 and 149.96, respectively, as well as an average of three comorbid psychiatric diagnoses. If the case is that those who improve the most attribute greater value to treatment and tend to stay engaged, this could explain differences with studies that looked at severity during the course or at the end of treatment. There could also be DBH-specific factors that explain possible differences in findings. DBH emphasizes transitioning patients to higher levels of care as a function of clinical needs observed at lower levels. This may allow for directing patients to levels of care needed to maintain engagement over time. Future research should investigate the effect of triage to higher levels of care over time on subsequent care completion rates.

### 4.2. Implications for practice

Our findings have several implications for practice. First, since we find that in our study patients with higher baseline depression severity and lower recovery are more likely to stay in treatment than patients with lower severity and higher initial recovery, interventions should be tailored to ensure that patients with lower severity and greater recovery receive adequate support and encouragement to stay in treatment. This might include showing patients changes in their PHQ-9 and RAS scores throughout their care to demonstrate their improvement over time.

The five factors for PHQ-9 and RAS may also help identify patients at high risk of dropout. Those with the lowest levels of suicidal ideation and thoughts of self-harm and the highest willingness to ask for help could be targeted with engagement retention interventions even if their aggregate scores suggest they are at lower risk of treatment dropout.

Engagement improvement efforts should also be concentrated on subgroups where the strongest associations between each measure and dropout were identified, particularly mental health, adolescents, and patients treated in the intermediate level of care setting. DBH has updated its patient dashboard to carefully monitor these at-risk groups, speaking to the importance of developing new knowledge on subgroups likely to dropout of treatment. For subgroups where no association between PHQ-9 or RAS was found (i.e., substance use disorder and eating disorder), new measures that relate more closely to the experiences of these patients will be needed to understand how condition-specific severity and recovery are associated with treatment retention.

New research on mechanisms driving these results is needed. For example, patients with greater baseline depression severity and lower recovery might be more likely to feel improvements in their health and perceive that their care is high quality, motivating retention. Patients with greater severity and lower recovery may also perceive they need to remain engaged in treatment to avoid engaging in harmful behavior. Similarly, patients with lower severity could be further along in their recovery, and could, therefore, be less inclined to continue receiving treatment. Future research should link care practices, quality, and changes in severity and recovery to treatment dropout.

Our results also call for expansions in patient-reported outcome measurement in behavioral health. Given the relationships between our outcome measures and treatment engagement, broader measurement-based care adoption could help ensure that patients at risk of treatment dropout remain in care. More information regarding patient severity and recovery could also be leveraged in decision-making around moving patients up or down levels of care and adjusting lengths of stay based on medical necessity criteria. The dropout frequency observed in our data may also speak to health disparities in care access. Specifically, we observe significant rates of patient-related dropout, which could be for reasons related to issues with consistently accessing services. Future research should confirm the association between dropout and care access issues in this setting, which, if identified, could be addressed by increasing virtual care offerings or strengthening relationships with urgent care centers.

Finally, in response to our findings, DBH is changing the way they define discharge categories by adding additional subcategories for patient-related and administrative treatment dropout. This will allowfor future research that can provide deeper clarity and actionable insight for both voluntary and involuntary discharge types.

### 4.3. Limitations

This study has several limitations. First, patients who take baseline assessments of their PHQ-9 and RAS may differ characteristically from patients who do not. Second, our estimates are associations; unobserved characteristics not included in the model may lead to residual confounding. Third, all measures are patient-reported and limited to individual items included in each measure, which may not reflect all relevant factors of depression severity and recovery. Lastly, patients at DBH may differ characteristically from patients treated by different providers. Findings may not necessarily generalize outside of this behavioral health care organization.

Despite limitations, the study also has several strengths. Our study includes a large sample size of nearly 15,000 patients who were administered the PHQ-9 and RAS at baseline. Our data include rich information on patient clinical characteristics and comorbidities, which are used to adjust for possible confounders in our regression models that might otherwise bias our results. We also have individual item responses for each measure in addition to aggregate scores, allowing us to drill into particular domains that drive our primary results (e.g., suicide items in the PHQ-9). Finally, our use of Cox proportional hazards models allows us to study risk of dropout while being cognizant of variation in patient length of stay.

### 4.4. Grant funding information

This research did not receive grant funding.

## 5. Conclusion

Greater PHQ-9 measured depression severity at baseline, and lower baseline RAS recovery scores were associated with a decreased likelihood of patient treatment dropout. Moreover, patients with greater levels of thoughts of dying and self-harm, self-attitude, and depression severity were least likely to drop out of treatment, while patients with greater willingness to ask for help, not dominated by symptoms and goal/success orientation, were the most likely to drop out of treatment. Interventions that engage less severe and more recovered patients in care to clinical completion may help improve their health outcomes. Increasing patient-reported outcomes measurement nationwide may help to ensure treatments are targeted appropriately.

## Supplementary Material

Multimedia component 1.

## Figures and Tables

**Table 1:** Characteristics of discovery behavioral health patients – all dropout.

Measure Description	Full Sample	Non-Dropout	Dropout	Standardized Difference

**Patient Level Measures**				
**Total Number of**	14689	10822	3867	-
**Patients - N (%)**	(100.00)	(73.67)	(26.33)	
**Age Group - N (%)**				
*Adolescent*	6241	4657	1584	−0.042
	(42.49)	(43.03)	(40.96)	
*Adult*	8448	6165	2283	0.042
	(57.51)	(56.97)	(59.04)	
**Sex - N (%)**				
*Female*	9695	7096	2599	0.035
	(66.00)	(65.57)	(67.21)	
*Male*	4994	3726	1268	−0.035
	(34.00)	(34.43)	(32.79)	
**Ethnicity - N (%)**				
*Hispanic or Latino*	2340	1700	640	0.023
	(15.93)	(15.71)	(16.55)	
*Not Hispanic or Latino*	9253	6715	2538	0.075
	(62.99)	(62.05)	(65.63)	
*Unknown Ethnicity*	3096	2407	689	−0.111
	(21.08)	(22.24)	(17.82)	
**Race - N (%)**				
*American Indian or*	100	73 (0.67)	27 (0.70)	0.004
*Alaska Native*	(0.68)			
*Asian*	544	427	117	−0.050
	(3.70)	(3.95)	(3.03)	
*Black or African*	780	574	206	0.001
*American*	(5.31)	(5.30)	(5.33)	
*Multi-Racial*	1126	819	307	0.014
	(7.67)	(7.57)	(7.94)	
*Native Hawaiian or*	54 (0.37)	36 (0.33)	18 (0.47)	0.022
*Pacific Islander*				
*Other Race*	231	158	73 (1.89)	0.034
	(1.57)	(1.46)		
*Unknown Race*	1661	1202	459	0.024
	(11.31)	(11.11)	(11.87)	
*White*	10193	7533	2660	−0.018
	(69.39)	(69.61)	(68.79)	
**Line of Service - N (%)**				
*Eating Disorders*	3928	2794	1134	0.079
	(26.74)	(25.82)	(29.33)	
*Mental Health*	5207	3768	1439	0.050
	(35.45)	(34.82)	(37.21)	
*Substance Use*	5554	4260	1294	−0.123
*Disorder*	(37.81)	(39.36)	(33.46)	
**Eating Disorders Diagnoses - N (%)**				
*Anorexia Nervosa*	2540	1838	702	0.031
	(17.29)	(16.98)	(18.15)	
*Avoidant Restrictive*	235	163	72 (1.86)	0.027
*Food Intake Disorder*	(1.60)	(1.51)		
*Binge Eating Disorder*	172	113	59 (1.53)	0.044
	(1.17)	(1.04)		
*Bulimia Nervosa*	327	215	112	0.059
	(2.23)	(1.99)	(2.90)	
*Other Specified*	652	463	189	0.029
*Feeding or Eating*	(4.44)	(4.28)	(4.89)	
*Disorder*				
**Substance Use Disorder Diagnoses - N (%)**				
*Alcohol Use Disorder*	3149	2528	621	−0.184
	(21.44)	(23.36)	(16.06)	
*Cannabis Use Disorder*	201	155	46 (1.19)	−0.021
	(1.37)	(1.43)		
*Cocaine Use Disorder*	185	143	42 (1.09)	−0.021
	(1.26)	(1.32)		
*Opioid Use Disorder*	815	577	238	0.035
	(5.55)	(5.33)	(6.15)	
*Sedative-Hypnotic Use*	235	159	76 (1.97)	0.039
*Disorder*	(1.60)	(1.47)		
*Unspecified Stimulant*	362	256	106	0.024
*Use Disorder*	(2.46)	(2.37)	(2.74)	
**Mental Health Disorder Diagnoses - N (%)**				
*Anxiety Disorder*	723	515	208	0.028
	(4.92)	(4.76)	(5.38)	
*Bipolar Disorder*	533	376	157	0.031
*Depression*	(3.63) 3713	(3.47) 2719	(4.06) 994	0.013
*Post-Traumatic Stress*	(25.28) 265	(25.12) 190	(25.70) 75 (1.94)	0.013
*Disorder* **Number of Diagnoses**	(1.80) 3.01	(1.76) 3.02	2.98	−0.030
**- Mean (Stddev)**	(1.41)	(1.42)	(1.40)	
**Episode Level Measures** **Total Number of**	17580	12983	4597	–
**Treatment Episodes -**	(100.00)	(73.85)	(26.15)	
**N (%)** **Care Setting - N (%)** *Intermediate Level of*	7566	5326	2240	0.156
*Care*	(43.04)	(41.02)	(48.73)	
*Residential*	10014 (56.96)	7657 (58.98)	2357 (51.27)	−0.156
**Length of stay - Mean**	38.07	42.81	24.68	−0.630
**(Stddev)**	(30.92)	(31.20)	(25.78)	
**Treatment Episodes**	1.26	1.27	1.25	−0.030
**per Patient - Mean** **(Stddev)**	(0.60)	(0.61)	(0.57)	
**PHQ-9 at Admission -**	13.29	13.50	12.69	−0.110
**Mean (Stddev)**	(7.43)	(7.37)	(7.56)	
**RAS at Admission -**	149.96	149.23	152.02	0.110
**Mean (Stddev)**	(24.50)	(24.31)	(24.91)	

Notes: The table displays characteristics of the analytic cohort for all variables included in the analyses and the breakdown between patients who drop out of treatment for any reason and non-dropout patients. The intermediate level of care reflects outpatient, intensive outpatient, and partial hospitalization. All estimates are presented as Hazard Ratios with 95% confidence intervals. Source: Discovery Behavioral Health Electronic Health Records Data 2021–2022.

## Appendix A. Supplementary data

Supplementary data to this article can be found online at https://doi.org/10.1016/j.psycom.2024.100185.
