# Supplementary material for: Association between depression severity, mental health recovery and dropout from behavioral health care treatment
Source: Psychiatry Res Commun. Author manuscript; Available in PMC 2026 Sep 15. (PMC13574114; doi:10.1016/j.psycom.2024.100185)
Supplement: Supplementary file 1 [file NIHMS2199492-supplement-Multimedia_component_1_.docx]

**Online Appendix**

**Appendix Table 1. Association between PHQ-9 and All Treatment Dropout by Length of Stay (0-30 days, 31-60 days, and 60+ days)**

| **Patient Sample** | **0-30 Days** | **31-60 Days** | **60+ days** |
| --- | --- | --- | --- |
| Full Sample | 0.990(0.986-0.995) | 0.989(0.980-0.999) | 0.994(0.981-1.008) |
| Eating Disorder Subgroup | Inestimable | 1.001(0.986-1.016) | 0.998(0.978-1.018) |
| Mental Health Subgroup | 0.985(0.976-0.993) | 0.994(0.979-1.010) | 0.987(0.965-1.011) |
| Substance Use Disorder Subgroup | 1.000(0.992-1.007) | 0.960(0.933-0.987) | 1.008(0.963-1.055) |
| Adolescent Subgroup | 0.984(0.976-0.992) | 0.993(0.980-1.006) | 0.984(0.964-1.004) |
| Adult Subgroup | 0.996(0.991-1.002) | 0.989(0.975-1.003) | 1.003(0.983-1.023) |
| Outpatient Subgroup | 0.982(0.974-0.990) | 1.000(0.988-1.012) | 0.995(0.980-1.010) |
| Residential Subgroup | 0.998(0.993-1.004) | 0.975(0.960-0.991) | 0.999(0.948-1.053) |

Notes: The table displays model estimates from the PHQ-9 treatment dropout analyses stratified by length of stay (0-30 days, 31-60 days, and 60+ days). The primary outcome is dropout for any reason, and each model includes covariate adjustment. Models were run for the full sample and cohort subgroups. The intermediate level of care reflects outpatient, intensive outpatient, and partial hospitalization. All estimates are presented as Hazard Ratios with 95% confidence intervals.

Source: Discovery Behavioral Health Electronic Health Records Data 2021-2022.

**Appendix Table 2. Association between RAS and All Treatment Dropout by Length of Stay (0-30 days, 31-60 days, and 60+ days)**

| **Patient Sample** | **0-30 Days** | **31-60 Days** | **60+ days** |
| --- | --- | --- | --- |
| Full Sample | 1.004(1.002-1.005) | 1.006(1.003-1.009) | 1.001(0.996-1.006) |
| Eating Disorder Subgroup | Inestimable | 1.003(0.998-1.009) | Inestimable |
| Mental Health Subgroup | 1.004(1.002-1.007) | 1.007(1.003-1.012) | 0.999(0.991-1.008) |
| Substance Use Disorder Subgroup | 1.002(1.000-1.005) | 1.004(0.994-1.013) | 0.996(0.981-1.012) |
| Adolescent Subgroup | 1.004(1.002-1.007) | Inestimable | 1.003(0.996-1.010) |
| Adult Subgroup | 1.002(1.000-1.004) | 1.002(0.997-1.007) | 0.999(0.991-1.006) |
| Outpatient Subgroup | 1.006(1.004-1.008) | 1.004(1.000-1.008) | 0.999(0.994-1.005) |
| Residential Subgroup | 1.002(1.000-1.004) | 1.009(1.003-1.014) | Inestimable |

Notes: The table displays model estimates from the RAS treatment dropout analyses stratified by length of stay (0-30 days, 31-60 days, and 60+ days). The primary outcome is dropout for any reason, and each model includes covariate adjustment. Models were run for the total sample and cohort subgroups. The intermediate level of care reflects outpatient, intensive outpatient, and partial hospitalization. All estimates are presented as Hazard Ratios with 95% confidence intervals.

Source: Discovery Behavioral Health Electronic Health Records Data 2021-2022.

**Appendix Table 3. Characteristics of Discovery Behavioral Health Patients – Patient & Administrative Dropout**

| **Measure Description** | **Non-Dropout** | **Patient Dropout** | **Standardized Difference** | **Administrative Dropout** | **Standardized Difference** |  |
| --- | --- | --- | --- | --- | --- | --- |
|  |  |  |  |  |  |  |
| **Patient Level Measures** |  |  |  |  |  |  |
| **Total Number of Patients - N (%)** | 10822 | 2234 | - | 1633 | - |  |
| **Age Group - N (%)** |  |  |  |  |  |  |
| *Adolescent* | 4657(43.03) | 790(35.36) | -0.1576 | 794(48.62) | 0.1124 |  |
| *Adult* | 6165(56.97) | 1444(64.64) | 0.1576 | 839(51.38) | -0.1124 |  |
| **Sex Assigned at Birth - N (%)** |  |  |  |  |  |  |
| *Female* | 7096(65.57) | 1495(66.92) | 0.0286 | 1104(67.61) | 0.0433 |  |
| *Male* | 3726(34.43) | 739(33.08) | -0.0286 | 529(32.39) | -0.0433 |  |
| **Ethnicity - N (%)** |  |  |  |  |  |  |
| *Hispanic or Latino* | 1700(15.71) | 345(15.44) | -0.0074 | 295(18.06) | 0.0628 |  |
| *Not Hispanic or Latino* | 6715(62.05) | 1468(65.71) | 0.0763 | 1070(65.52) | 0.0722 |  |
| *Unknown Ethnicity* | 2407(22.24) | 421(18.85) | -0.0840 | 268(16.41) | -0.1481 |  |
| **Race - N (%)** |  |  |  |  |  |  |
| *American Indian or Alaska Native* | 73(0.67) | 14(0.63) | -0.0050 | 13(0.80) | 0.0152 |  |
| *Asian* | 427(3.95) | 68(3.04) | -0.0496 | 49(3.00) | -0.0519 |  |
| *Black or African-American* | 574(5.30) | 119(5.33) | 0.0013 | 87(5.33) | 0.0013 |  |
| *Multi-Racial* | 819(7.57) | 170(7.61) | 0.0015 | 137(8.39) | 0.0303 |  |
| *Native Hawaiian or Pacific Islander* | 36(0.33) | 9(0.40) | 0.0116 | 9(0.55) | 0.0332 |  |
| *Other Race* | 158(1.46) | 39(1.75) | 0.0231 | 34(2.08) | 0.0470 |  |
| *Unknown Race* | 1202(11.11) | 244(10.92) | -0.0061 | 215(13.17) | 0.0631 |  |
| *White* | 7533(69.61) | 1571(70.32) | 0.0155 | 1089(66.69) | -0.0627 |  |
| **Line of Service - N (%)** |  |  |  |  |  |  |
| *Eating Disorders* | 2794(25.82) | 698(31.24) | 0.1202 | 436(26.70) | 0.0200 |  |
| *Mental Health* | 3768(34.82) | 730(32.68) | -0.0453 | 709(43.42) | 0.1769 |  |
| *Substance Use Disorder* | 4260(39.36) | 806(36.08) | -0.0677 | 488(29.88) | -0.2003 |  |
| **Eating Disorders Diagnoses - N (%)** |  |  |  |  |  |  |
| *Anorexia Nervosa* | 1838(16.98) | 442(19.79) | 0.0726 | 260(15.92) | -0.0286 |  |
| *Avoidant Restrictive Food Intake Disorder* | 163(1.51) | 45(2.01) | 0.0380 | 27(1.65) | 0.0112 |  |
| *Binge Eating Disorder* | 113(1.04) | 26(1.16) | 0.0115 | 33(2.02) | 0.0799 |  |
| *Bulimia Nervosa* | 215(1.99) | 77(3.45) | 0.0898 | 35(2.14) | 0.0105 |  |
| *Other Specified Feeding or Eating Disorders* | 463(4.28) | 108(4.83) | 0.0264 | 81(4.96) | 0.0324 |  |
| **Substance Use Disorder Diagnoses - N (%)** |  |  |  |  |  |  |
| *Alcohol Use Disorder* | 2528(23.36) | 406(18.17) | -0.1282 | 215(13.17) | -0.2661 |  |
| *Cannabis Use Disorder* | 155(1.43) | 22(0.98) | -0.0413 | 24(1.47) | 0.0033 |  |
| *Cocaine Use Disorder* | 143(1.32) | 27(1.21) | -0.0098 | 15(0.92) | -0.0380 |  |
| *Opioid Use Disorder* | 577(5.33) | 136(6.09) | 0.0328 | 102(6.25) | 0.0394 |  |
| *Sedative-Hypnotic Use Disorder* | 159(1.47) | 51(2.28) | 0.0597 | 25(1.53) | 0.0049 |  |
| *Unspecified Stimulant Use Disorder* | 256(2.37) | 72(3.22) | 0.0516 | 34(2.08) | -0.0197 |  |
| **Mental Health Disorder Diagnoses - N (%)** |  |  |  |  |  |  |
| *Anxiety Disorder* | 515(4.76) | 113(5.06) | 0.0139 | 95(5.82) | 0.0474 |  |
| *Bipolar Disorder* | 376(3.47) | 89(3.98) | 0.0269 | 68(4.16) | 0.0360 |  |
| *Depression* | 2719(25.12) | 510(22.83) | -0.0537 | 484(29.64) | 0.1015 |  |
| *Post-Traumatic Stress Disorder* | 190(1.76) | 44(1.97) | 0.0155 | 31(1.90) | 0.0104 |  |
| **Number of Diagnoses - Mean (Stddev)** | 3.02(1.42) | 2.94(1.39) | -0.0600 | 3.03(1.41) | 0.0100 |  |
| **Episode Level Measures** |  |  |  |  |  |  |
| **Total Number of Treatment Episodes - N (%)** | 12983 | 2614 | - | 1983 | - |  |
| **Care Setting - N (%)** |  |  |  |  |  |  |
| *Intermediate level of care* | 5326(41.02) | 1392(53.25) | 0.2469 | 848(42.76) | 0.0353 |  |
| *Residential* | 7657(58.98) | 1222(46.75) | -0.2469 | 1135(57.24) | -0.0353 |  |
| **Length of stay - Mean (Stddev)** | 42.81(31.20) | 22.48(26.01) | -0.7100 | 27.59(25.19) | -0.5400 |  |
| **Treatment Episodes per Patient - Mean (Stddev)** | 1.27(0.61) | 1.22(0.53) | -0.0900 | 1.28(0.62) | 0.0200 |  |
| **PHQ-9 at Admission - Mean (Stddev)** | 13.50(7.37) | 12.64(7.58) | -0.1200 | 12.77(7.54) | -0.1000 |  |
| **RAS at Admission - Mean (Stddev)** | 149.23(24.31) | 152.86(24.58) | 0.1500 | 150.91(25.31) | 0.0700 |  |

Notes: The table displays characteristics of the analytic cohort for all variables included in the analyses and the breakdown between patients who drop out of treatment for patient and administrative reasons versus non-dropout patients. The intermediate level of care reflects outpatient, intensive outpatient, and partial hospitalization. All estimates are presented as Hazard Ratios with 95% confidence intervals.

Source: Discovery Behavioral Health Electronic Health Records Data 2021-2022.
